# Ultrasound-guided perineural intercostal autologous platelet-rich plasma in the treatment of chronic post-thoracotomy pain syndrome – A prospective case series

**DOI:** 10.1016/j.inpm.2024.100448

**Published:** 2024-11-16

**Authors:** César Gracia-Fabre, Tomas Cuñat, Eduardo Matos-Ribeiro, Rosario Armand-Ugon, Guilherme Ferreira-Dos-Santos

**Affiliations:** a Department of Anesthesiology, Critical Care, and Pain Medicine, Consorci Sanitari Integral de Barcelona, Barcelona, Spain; b Division of Pain Medicine, Department of Anesthesiology, Reanimation, and Pain Medicine, Hospital Clínic de Barcelona, University of Barcelona, Barcelona, Spain; c Department of Anesthesiology, Unidade Local de Saúde de Lisboa Ocidental, Lisboa, Portugal; d Fundación MIVI, Barcelona, Spain

**Keywords:** Chronic Post-Thoracotomy Pain Syndrome, Intercostal Nerve Block, Platelet-Rich Plasma, Regenerative Medicine, Ultrasound

## Abstract

**Background:**

Post-thoracotomy pain syndrome poses a significant challenge in clinical management due to its debilitating nature. Current treatment strategies often involve multimodal approaches, including pharmacology and interventional procedures. Recently, platelet-rich plasma has emerged as a potential therapeutic option for chronic neuropathic pain, yet its efficacy in post-thoracotomy pain syndrome remains unexplored.

**Methods:**

This prospective consecutive case series aimed to evaluate the effectiveness of autologous platelet-rich plasma in alleviating chronic post-thoracotomy pain syndrome. Ten patients with persistent thoracic post-surgical pain were consecutively recruited. Platelet-rich plasma was administered via ultrasound-guided perineural intercostal injections. Pain intensity, opioid consumption, and quality of life were assessed pre-treatment and at one- and three-month follow-ups.

**Results:**

Platelet-rich plasma administration led to a significant reduction in pain intensity, with median Numerical Rating Scale scores decreasing from 8.5 to 3.0 at one month and 4.0 at three months post-treatment. At one month, 90 % of patients achieved a reduction in NRS scores exceeding the minimal clinically important difference (95 % CI: 71 %, 109 %), and this proportion was maintained at three months. Although opioid consumption showed a downward trend, it did not reach statistical significance. Improvements were observed in the EQ-5D-3L index and visual analogue scale scores, indicating enhanced quality of life post-treatment.

**Conclusions:**

This prospective consecutive case series suggests that autologous platelet-rich plasma may offer a promising adjunctive therapy for chronic post-thoracotomy pain syndrome. However, limitations including the lack of a control group and small sample size underscore the need for further research to establish the efficacy and optimize the application of platelet-rich plasma in managing post-thoracotomy pain syndrome.

## 1 Background

Chronic postsurgical pain is defined as pain that emerges or worsens following surgical intervention and continues even after the typical healing period, persisting for more than three months post-surgery [[1], [2], [3]]. Previous studies in the literature have reported an incidence of up to 50 % of post-thoracotomy pain following thoracic or chest wall surgery. While nearly half of these patients present with neuropathic pain, post-thoracotomy pain is thought to be multifaceted, extending beyond a purely neuropathic dimension [3].

On presentation, patients usually describe pain localized to the chest wall, closely linked to the surgical area and scar, often worsening with movement. Accompanying sensory changes near the scar are often present and suggest intercostal nerve injury as a key etiological factor [3]. While appropriately treated acute post-thoracotomy pain often resolves, a significant number of patients develop post-thoracotomy pain syndrome (PTPS), with up to 65 % of patients experiencing some pain and 10 % suffering life-altering, debilitating pain [4].

Current clinical data and international guidelines published in the literature suggest that adequate management of PTPS requires a multimodal treatment strategy, including a combination of pharmacology, psychological support and interventional pain procedures [[3], [4], [5]]. Considering the latter, two recent studies by Guerra-Londono *et al*. (2021) and Elkhashab *et al*. (2021) showed that intercostal nerve blocks performed under ultrasound (US) guidance may play a key role in managing both acute and chronic post-thoracotomy pain [5,6]. In their study, Guerra-Londono *et al*. (2021) showed that the efficacy of intercostals nerve blocks was comparable to that of thoracic epidural injections and paravertebral blocks for post-thoracotomy pain [5].

Recently, perineural platelet-rich plasma (PRP) has been gaining prominence in the management of peripheral neuropathic pain, due to its anti-inflammatory and analgesic properties. Preclinical studies highlight its ability to alleviate allodynia by modulating cytokine levels and disrupting key pain pathways, like the p38 mitogen-activated protein kinase [7].

Clinically, while PRP has been investigated in the treatment of peripheral neuropathic pain conditions such as radiculopathy – with applications ranging from epidural to perineural nerve root injections – the current evidence is limited, highlighting the need for further research to confirm its efficacy in achieving significant pain reduction. Additionally, its use during surgeries may suggest a potential dual role in pain relief and prevention of neuropathic pain onset [7]. Therefore, PRP stands out as a potentially promising option in the management of chronic neuropathic pain of peripheral origin, including post-thoracotomy pain syndrome.

In this prospective consecutive case series of 10 patients, we aimed to assess the effectiveness of autologous PRP in alleviating chronic post-thoracotomy pain. We hypothesized that a single perineural application of autologous PRP targeting the intercostal nerves would provide clinically significant pain relief. Clinical efficacy was assessed through measures of pain intensity, opioid use, and patient-reported quality of life.

## 2 Methodology

This study was performed at the Division of Pain Medicine at Hospital Clínic de Barcelona (HCB), University of Barcelona (Spain). Institutional Review Board (IRB) (HCB/2022/0610) approval was granted before patient recruitment began. This research was conducted following the STROBE guidelines (STrengthening the Reporting of Observational studies in Epidemiology. 10 patients were recruited consecutively at the Division of Pain Medicine in accordance with the following inclusion and exclusion criteria:

*Inclusion*: Adult patients (>18 years-old) presenting with thoracic post-surgical pain for more than three months; average daily pain rated as intense, with a score of 7 or above in the Numerical Rating Scale (NRS); a score of 4 or more in the Spanish validated version of the *Douleur Neuropathique* 4 (DN4) questionnaire, indicating a highly likely component of neuropathic pain [8]; insufficient self-reported pain relief (<30 %) from at least three different anti-neuropathic pain medication agents.

*Exclusion*: Active local infection in the thoracic or chest wall region; active oncologic disease at the time of recruitment; intercostal nerve block with corticosteroids in the 3 months prior to recruitment; known hematologic condition with influence on platelet number or platelet activation; pregnancy or lactation.

**Patient Demographics and Clinical Profile:** Data was compiled at the time of recruitment. Data collected included: age; sex; body mass index (BMI); and medical diagnosis that lead to thoracotomy. Pain severity and its impact on quality of life was assessed by the following data: anti-neuropathic pain medication agents tried; daily consumption of opioids measured in oral morphine equivalents (OME); DN4, PainDETECT, and the EQ-5D-3L questionnaires. The EQ-5D-3L index was employed to quantitatively evaluate the patient's health status across five dimensions: mobility, self-care, usual activities, pain/discomfort, and anxiety/depression, each with three levels of severity.

**Collection and Preparation of PRP:** PRP was collected and prepared utilizing the 3E-20 PRP System (Pervice Korea Co., Ltd, Seoul, South Korea) in strict accordance with the manufacturer's technical description: 3.0 mL of anticoagulant citrate dextrose solution A (ACD-A) were utilized for collection of 17 mL of whole blood. The 20 mL solution was then centrifuged at 3000 revolutions per minute (RPM) for 5 min. After centrifugation, PRP was collected using a “push-up” extraction method without needle (closed system) in a 5 mL syringe, drawing 3 mL of PRP concentrate containing the buffy coat. Final platelet concentration utilizing the above-mentioned method has been calculated to be between 9.3 and 11.5 times greater than baseline platelet concentration [9]. Prior to administration, the final PRP concentrate in the 5 mL syringe was externally activated using 1.0 mL of calcium chloride. The final PRP concentrate was not analyzed using an automated cell counter tool prior to administration. The platelet-poor plasma fraction was discarded.

**Fig. 1:**
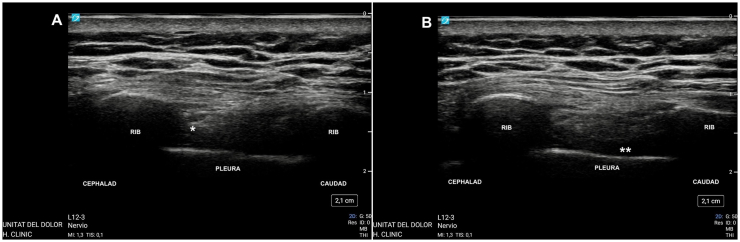
A) Ultrasound image showing the final position of the needle tip using an in-plane technique. The tip is adequately positioned between the internal and innermost intercostal muscles at the inferior border of the rib of the target level; B) Ultrasound image immediately after perineural administration of platelet-rich plasma, illustrating the visualization of pleural edge compression, confirming adequate spread of the platelet-rich plasma concentrate.

**Ultrasound-Guided Perineural Intercostal PRP Administration:** 3 mL of PRP was administered under real-time ultrasound-guidance, using a Sonosite PX Ultrasound System (FUJIFILM SonoSite, Inc., Bothell, Washington, United States of America) equipped with an L12-3 MHz linear array transducer. The procedure was performed using an in-plane technique in a caudad-to-cephalad approach in the longitudinal plane, with a 22G 50 mm Stimuplex Ultra 360 needle (B Braun Medical AG, Melsungen, Germany), with direct visualization of the needle tip final location at the inferior border of the rib of the target level, between the internal and innermost intercostal muscles (Fig. 1A–B) [10]. Proximity to the target intercostal nerve was confirmed by positive sensory stimulation at <0.7 mA.

**Patient Follow-Up After Treatment:** Follow-up assessments were performed at one and three months after treatment. Data collected at these assessments was aimed at tracking changes in pain intensity in the NRS, daily opioid medication use in OME and the EQ-5D-3L index, with the latter being recorded specifically at the three-month mark to assess long-term health-related quality of life improvements. We defined a minimal clinically important difference (MCID) in the NRS as a reduction of 3 points.

**Statistical Analysis:** Sample size calculation was not performed due to the nature of this study. Data analysis was conducted using RStudio (Posit Software, Inc., Boston, Massachusetts, United States of America) version 2023.09.1. We employed a non-parametric approach for paired data analysis to discern the effects of the PRP treatment. Specifically, we utilized the Wilcoxon signed-rank test to evaluate the changes

## 3 Results

From January to June of 2022, ten patients were consecutively recruited at the Division of Pain Medicine at HCB. All patients met the inclusion criteria, presenting with thoracic post-surgical pain lasting for more than three months. Data on patient demographics, pain severity and its impact on quality of life at baseline is presented in Table 1.

**Table 1:** Patient demographics and clinical profile. Values are presented as median [IQR - interquartile range] or “n” and percentage (%). *Legend:* BMI - Body Mass Index; DN4 - Douleur Neuropathique 4 Questionnaire; EQ-5D-3L - EuroQol 5-Dimensions 3-Levels Questionnarie; NRS- Numeric Rating Scale; OME - Oral Morphine Equivalents; VAS - Visual Analogue Scale.

Variable	Median (IQR) or n (%)
Age (years)	57.0 (54.0–59.0)
BMI (kg/m^2^)	22.5 (21.0–24.75)
Sex (female)	6 (60 %)
Psychiatric Comorbidities	5 (50 %)
Indication for Surgery	
*Non-small cell lung cancer*	6 (60 %)
*Benign Neurogenic Tumor*	3 (30 %)
*Infection*	1 (10 %)
DN4	6.5 [6,7]
PainDETECT	19.5 (16.75–20.0)
EQ-5D-3L index	0.238 [0.191–0.353]
EQ-5D-3L VAS	30.0 (20.0–53.75)
Pre treatment NRS	8.5 (6.25–9.0)
Pre treatment OME (mg)	25.0 (5.0–55.0)
Postoperative Acute Pain	1 (10 %)
Surgical Complications	3 (30 %)
Duration of Thoracic Pain (months)	8 (4.5–14.75)
Type of Pain	
*Likely neuropathic pain*	9 (90 %)
*Likely* m*ixed pain*	1 (10 %)
Pain Location	
*Anterior*	7 (70 %)
*Lateral*	9 (90 %)
*Posterior*	6 (60 %)
*1-*7th *ribs*	8 (80 %)
*8-*10th *ribs*	1 (10 %)

**Fig. 2:**
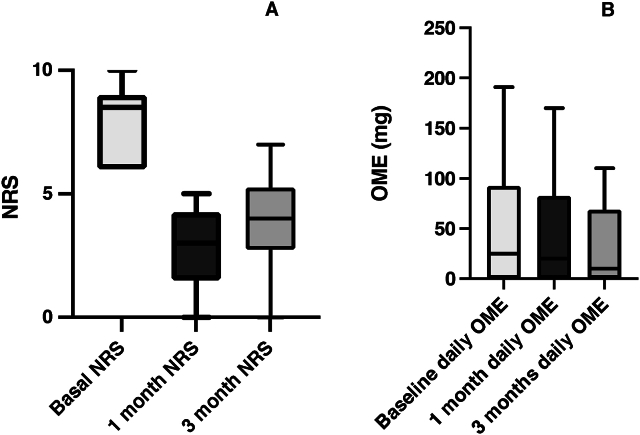
A) Numerical rating scale scores at baseline, 1 month post procedure and 3 months post procedure; B) Daily oral morphine equivalents consumption at baseline, 1 month post procedure and 3 months post procedure. Legend: NRS - Numeric rating scale; OME - Oral morphine equivalents.

The administration of perineural intercostal autologous PRP led to a significant reduction in NRS scores, decreasing from a baseline median of 8.5 (IQR 6.25–9.0) to 3.0 (IQR 2.0–4.0) at one month (p = 0.006, 95 % CI: 4 %, 6 %) and 4.0 (IQR 3.0–4.75) at three months (p = 0.005, 95 % CI: 3 %, 6 %), as shown in Fig. 2-A. At one month, 90 % of patients achieved a reduction in NRS scores exceeding the minimal clinically important difference (MCID) (95 % CI: 71 %, 109 %), and 90 % maintained this level of improvement at three months (95 % CI: 71 %, 109 %).

Regarding the consumption of daily OME, a downward trend was observed from an initial median dose of 25 mg (IQR 5–55 mg). At the one-month mark post-treatment, the median consumption decreased to 20 mg (IQR 0–55 mg), although this decrease did not reach statistical significance (p = 0.1814, 60 % CI [21.0, 40.0]). By the three-month interval, the median morphine equivalent consumption was further reduced to 10 mg (IQR 0–55 mg), with this reduction also not being statistically significant (p = 0.1003, 80 % CI [20.0, 96.0]). These findings can be seen in Fig. 2-B.

**Fig. 3:**
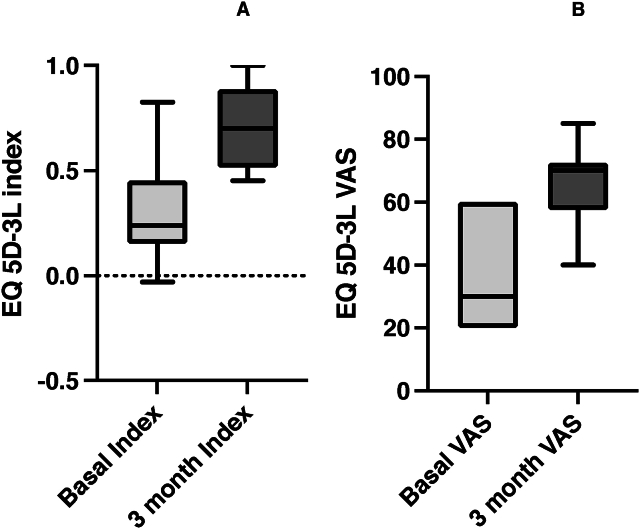
A) EQ-5D-3L index scores at baseline and 3 Months post procedure. EQ-5D-3L, EuroQol 5-dimension 3-level; B) EQ-5D-3L VAS scores at baseline and 3 Months post procedure. Legend: VAS - visual analogue scale.

An improvement was observed in both the EQ-5D-3L index and EQ-5D-3L VAS scores. For the EQ-5D-3L index, scores rose from a baseline median of 0.238 (IQR 0.191–0.353) to 0.701 (IQR 0.548–0.872) at the three-month mark (p = 0.009, 95 % CI [−0.539, −0.353]). Similarly, the EQ-5D-3L VAS scores also showed significant improvement, increasing from a baseline median of 30 (IQR 20.00–53.75) to 70 (IQR 62.5–70.0) in the same period (p = 0.009, 95 % CI [−47.5, −22.5]). These results are illustrated in Fig. 3A and B.

## 4 Discussion

This prospective consecutive case series represents a pioneering effort in documenting the effects of PRP concentrates in chronic post-thoracotomy pain. The significant reduction observed in NRS scores together with the improvement in EQ-5D-3L index and VAS scores at both one- and three-months post-treatment suggests that PRP could be an effective adjunct in the multimodal management of chronic post-thoracotomy pain.

Recently, the role of cytokines in the development and maintenance of chronic neuropathic pain conditions (including chronic post-thoracotomy pain), has been the subject of extensive study. Preclinical evidence indicates that cytokines secreted by immune and glial cells play a crucial role in the development of neuropathic pain conditions, as demonstrated by the effectiveness of tumor necrosis factor alfa (TNF-α) inhibitors and antibodies against interleukin 6 (IL-6) and IL-1β in animal models [11]. Clinical studies have corroborated these findings, demonstrating altered cytokine expression in patients with chronic neuropathic pain, with elevated pro-inflammatory cytokines and reduced anti-inflammatory cytokines observed in various tissues and fluids. This imbalance suggests the potential therapeutic targeting of cytokines in neuropathic pain management [[12], [13], [14]].

Over the last decade, PRP has shown promise in the treatment of chronic neuropathic pain. Recent preclinical studies have demonstrated that PRP induces a reduction in local concentration of pro-inflammatory cytokines like TNF-α and IL-1β [15]. Clinical studies have highlighted the effectiveness of PRP in the management of multiple types of chronic pain, including various forms of neuropathic pain such as mild-to-moderate carpal tunnel syndrome [[16], [17], [18]]. These studies suggest that the effects of PRP concentrates go beyond merely adjusting the local levels of cytokines; they may also involve direct modulation of pain pathways and enhancement of nerve function, contributing to its analgesic effects in neuropathic conditions [[16], [17], [18]]. Nonetheless, evidence is still scarce and more extensive research is warranted to ascertain the efficacy and safety profile of PRP concentrates in the treatment of chronic neuropathic pain of peripheral origin [17,18].

This study has several limitations which should be taken into consideration, including the case series design and the pre- and post-treatment data analysis, which, without a control group, introduce potential confounding factors and limit the attribution of outcomes solely to the therapeutic effect of PRP administration. The small sample size and lack of randomization constrain the generalizability and raise the risk of selection bias. Moreover, the tools used for assessment of clinical outcomes, such as the NRS and the EQ-5D-3L index and VAS scores, while standard, are subject to individual patient interpretation and may not fully encapsulate the multidimensional impact of chronic pain, necessitating cautious interpretation of the results and underscoring the need for broader, more controlled studies. Another notable limitation lies in the broader uncertainty surrounding clinical practices in the context of treating chronic post-thoracotomy pain. This includes the variability in PRP preparation methods (e.g., centrifugation technique, choice of anticoagulant, and activation method), the dynamics of platelet concentrations, and the nuances of PRP administration protocols (number and frequency of injections). These factors collectively contribute to the uncertainty in determining the optimal approach for PRP treatment. This lack of definitive knowledge about the most effective PRP protocol is a significant challenge and links to the broader limitations of our study, where the efficacy of our specific approach cannot be conclusively compared to other potential methods due to the absence of a standardized treatment model in this emerging field [[15], [16], [17], [18]].

## 5 Conclusion

This prospective consecutive case series provides encouraging initial evidence supporting the effectiveness of PRP concentrates in improving chronic neuropathic pain following thoracotomy. The significant reduction in pain intensity and improvements in quality-of-life scores post-treatment highlight the potential value of autologous PRP concentrates as an adjunct in the management of this debilitating condition. Nevertheless, further research with larger sample sizes and controlled study designs is warranted to fully elucidate role and optimize the application of autologous PRP in the management of chronic post-thoracotomy pain syndrome.

## Data availability statement

The data supporting the findings of this study are available on request from the corresponding author. The data are not publicly available due to privacy or ethical restrictions.

## Conflict of interest disclosure

The authors did not receive any direct or indirect financial benefits for the publication of this manuscript.

## Ethics approval statement

Institutional Review Board approval (HCB/2022/0610) was granted before patient recruitment began.

## Patient consent statement

Patient consent was obtained for participation in the study.

## Author contribution statement

CGF and TC were responsible for the design of the study and for recruitment and treatment of the participants. EMR and RAU reviewed the data collected and drafted the initial version of the manuscript. GFDS was responsible for the final version of the manuscript submitted for publication and for overseeing the study.

## Funding statement

No funding was received for the conduct of this study.

## Declaration of competing interest

The authors declare that they have no known competing financial interests or personal relationships that could have appeared to influence the work reported in this paper.
